# Transradial versus Transfemoral Approach in Patients Undergoing Percutaneous Coronary Intervention for Acute Coronary Syndrome. A Meta-Analysis and Trial Sequential Analysis of Randomized Controlled Trials

**DOI:** 10.1371/journal.pone.0096127

**Published:** 2014-05-12

**Authors:** Raffaele Piccolo, Gennaro Galasso, Ernesto Capuano, Stefania De Luca, Giovanni Esposito, Bruno Trimarco, Federico Piscione

**Affiliations:** 1 Department of Advanced Biomedical Sciences, Federico II University, Naples, Italy; 2 Department of Medicine and Surgery, University of Salerno, Salerno, Italy; Cliniche Humanitas Gavazzeni, Italy

## Abstract

**Background:**

Transfemoral approach (TFA) remains the most common vascular access for percutaneous coronary intervention (PCI) in many countries. However, in the last years several randomized trials compared transradial approach (TRA) with TFA in patients with acute coronary syndrome (ACS), but only few studies were powered to estimate rare events. The aim of the current study was to clarify whether TRA is superior to TFA approach in patients with ACS undergoing percutaneous coronary intervention. A meta-analysis, meta-regression and trial sequential analysis of safety and efficacy of TRA in ACS setting was performed.

**Methods and Results:**

Medline, the Cochrane Library, Scopus, scientific session abstracts and relevant websites were searched. Data concerning the study design, patient characteristics, risk of bias, and outcomes were extracted. The primary endpoint was death. Secondary endpoints were: major bleeding and vascular complications. Outcomes were assessed within 30 days. Eleven randomized trials involving 9,202 patients were included. Compared with TFA, TRA significantly reduced the risk of death (odds ratio [OR] 0.70; 95% confidence interval [CI], 0.53–0.94; p = 0.016), but this finding was not confirmed in trial sequential analysis, indicating that sufficient evidence had not been yet reached. Furthermore, TRA compared with TFA reduced the risk of major bleeding (OR 0.60; 95% CI, 0.41–0.88; p = 0.008) and vascular complications (OR 0.35; 95% CI, 0.28–0.46; p<0.001); these findings were supported by trial sequential analyses.

**Conclusions:**

In patients with ACS undergoing PCI, a lower risk of death was observed with TRA. Nevertheless, the association between mortality and TRA in ACS setting should be interpreted with caution because it is based on insufficient evidence. However, because of the clinical relevance associated with major bleeding and vascular complications reduction, TRA should be recommended as first-choice vascular access in patients with ACS undergoing cardiac catheterization.

## Introduction

Percutaneous coronary intervention (PCI) represents a cornerstone for the treatment of patients with acute coronary syndrome (ACS). Currently, transfemoral approach (TFA) is the most common access for PCI in many countries [1]. During the last two decades, transradial approach (TRA) emerged as a valid alternative to TFA, because of earlier ambulation, shorter hospital stay and possibly reduced bleeding risk [2]. Despite these advantages, TRA for catheterization was performed infrequently (<3%) in the United States between 2005 and 2009 [3]. The reasons of this uncommon use remain uncertain, but could include familiarity with TFA and concerns for the longer learning curve of TRA, along with increased radiation exposure [4]. In the last years, several randomized clinical trials compared these two approaches in patients with ACS, but only few studies were adequately powered to allow a reliable estimation of rare events. Furthermore, recent meta-analyses assessing the role of TRA in ACS setting excluded patients with non-ST-segment elevation myocardial infarction, which represents the most frequent ACS presentation [5], [6]. Therefore, the aim of the current study was to perform a meta-analysis and trial sequential analysis of randomized trials evaluating the clinical outcomes following TRA versus TFA across the whole spectrum of ACS.

## Methods

### Data sources and searches

We searched Medline, the Cochrane Library, Scopus, scientific session abstracts (published in Circulation, Journal of the American College of Cardiology, European Heart Journal and The American Journal of Cardiology), and relevant websites (www.acc.org, www.americanheart.org, www.europcronline.com, www.escardio.org, www.clinicaltrialresults.org, www.tctmd.com and www.theheart.org). The reference list of relevant studies was additionally scanned. No language, publication date, or publication status restrictions were imposed. The last search was run on 15^th^ June, 2013. The following search terms were matched: “femoral”, “radial”, “transradial”, “transfemoral”, “percutaneous coronary intervention”, “randomized”, “acute coronary syndrome”, “myocardial infarction”, “unstable angina”, “non-ST-segment elevation”, “ST-segment elevation”.

### Study selection

To be included, the citation had to meet the following criteria: 1) random treatment allocation; 2) inclusion of patients with ACS; and 3) the use of TRA in the experimental arm. Exclusion criteria were: 1) ongoing studies; 2) irretrievable data and 3) trials not reporting death occurrence during follow-up. Complete electronic search strategy for Medline (PubMed) and The Cochrane Library was reported in the Supporting Information.

### Data Extraction and Quality Assessment

Two investigators (R.P. and G.G.) independently assessed reports for eligibility at title and/or at abstract level, with divergences resolved with a third reviewer (F.P.); studies that met inclusion criteria were selected for further analysis. The risk of bias was evaluated by the same two reviewer authors, in accordance with The Cochrane Collaboration methods and considering the following methodological items: random sequence generation, allocation concealment, blinding of participants and personnel, blinding of outcome assessment, incomplete outcome data, selective reporting, other bias and sample size calculation. We did not use a quality score, since this practice has been previously discouraged [7].

The primary endpoint of this meta-analysis was death within 30 days. Secondary endpoints were: major bleeding and vascular complications. Per protocol definitions of clinical endpoints were reported in the Table S1 in File S1.

### Data Synthesis and Analysis

Statistical analysis was performed with STATA 11 statistical software (STATA Corp, College Station, Texas, USA). The к statistic was used to assess agreement between reviewers for study selection. Odds ratio (OR) and 95% confidence intervals (95% CI) were used as summary statistics. The pooled OR was calculated by using the fixed effects Mantel-Hænzel model, while, in case of significant heterogeneity across studies, the random effects DerSimonian and Laird model was reported instead. In case of statistical significance, the number needed to treat (NNT) and the number of avoided events per 1,000 treated patients were provided. The Breslow-Day chi-squared test was calculated to test the statistical evidence of heterogeneity across the studies (p<0.1). In addition, we used the I^2^ statistic, which describes the percentage variation across studies that is due to heterogeneity rather than chance. As a guide, I^2^ values <25% indicated low, 25–50% indicated moderate, and >50% indicated high heterogeneity [8]. The influence of single studies on the summary estimates was examined graphically by checking how the elimination of each study affected the resulting summary estimate of OR. We assessed the possibility of small-study effects by visual inspection of funnel plot asymmetry. Because graphical evaluation can be subjective, we performed both Harbord [9] and Peters tests [10], as formal statistical tests for publication bias. A weighted random-effect meta-regression analysis was used to evaluate relationship between the risk of study endpoints and the following study-level covariates: age, sex, year of publication, enrolling centres (single- vs. multi-centre), sample size (<150 patients vs. ≥150 patients), proportion of primary PCI, glycoprotein IIb/IIIa inhibitors use and crossover rates to TFA. Furthermore, the relationship between the magnitude of risk reduction with TRA and the baseline risk for bleeding/vascular complications was also investigated with the *metareg* command, as previously described [11].

Trial sequential analysis was performed according to the monitoring boundaries approach [12], [13], by using TSA version 0.9 beta (www.ctu.dk/tsa). This is a methodology that combines an a priori information size calculation for a meta-analysis with the adaptation of monitoring boundaries to evaluate the accumulating evidence [14]. The information size calculation is similar to the sample size calculation in a single trial, allowing a quantification of the reliability of cumulative data in meta-analyses. Trial sequential analysis was obtained with alfa set to 5%, power to 80% and including the control event proportion observed in the meta-analysis. For death, major bleeding and vascular complication we chose a 20%, 35% and 50% relative risk reduction, respectively.

The study was realized in compliance with the Preferred Reporting Items for Systematic reviews and Meta-Analyses (PRISMA) statement [15].

## Results

**Figure 1:**
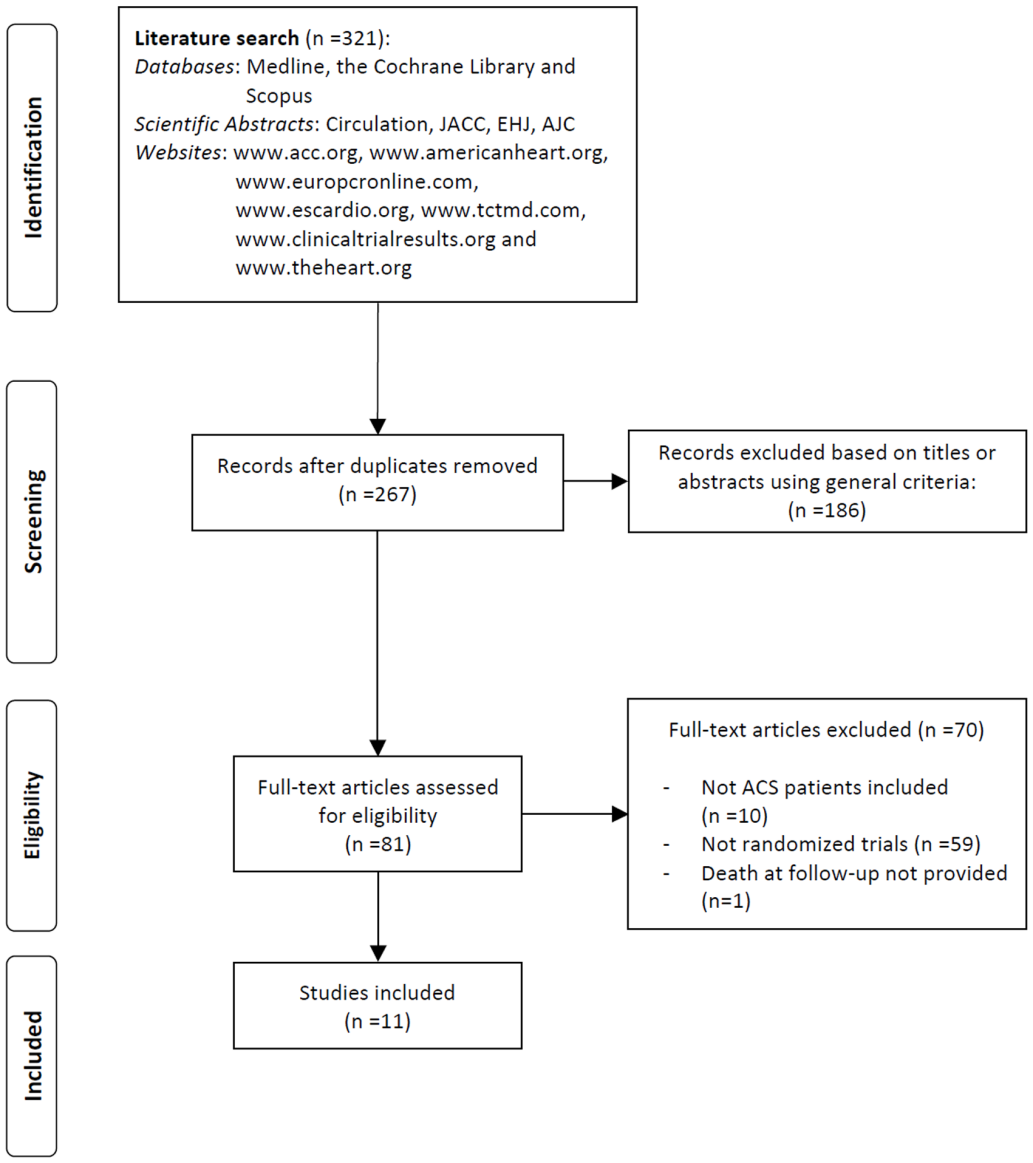
Flow diagram of trial selection. ACS, acute coronary syndrome.

As reported in Figure 1, we screened the title and/or the abstract of 321 potentially eligible publications. Of these, 240 citations were excluded because they were not relevant to this study or were duplicated publications. Eighty-one studies were thus assessed for eligibility and 70 records were discarded because the inclusion criteria were not met. Finally, eleven trials [16]–[26] were included in this meta-analysis, enrolling a total of 9,202 patients (4,583 or 49.3% randomly assigned to TRA and 4,619 or 50.7% randomly assigned to TFA). The interobserver agreement for study selection was very good, with κ = 0.95. The main characteristics of the included studies are summarized in Table 1. The risk of bias among studies is reported in Table 2. Anticoagulation was obtained with heparin in the vast majority of patients.

**Table 1:** Main Characteristics of included trials.

Trial	Year of publication	Period of enrollment	Multi-centre	Patients number		Mean age (years)		Male (%)		ACS type	Rescue PCI (%)		Follow-up	Gp IIb/IIIa inhibitors (%)		Type of Gp IIb/IIIa inhibitors	Hemostasis		Crossover (%)	
				TFA	TRA	TFA	TRA	TFA	TRA		TFA	TRA		TFA	TRA		TFA	TRA	TFA	TRA
**FARMI (16)**	2007	2004-05	No	57	57	60	58	86	82.5	STEMI	49.1	35.1	In-hospital	100	100	Abciximab	manual compression	manual compression	1.8	12.3
**Gan et al. (17)**	2009	2004-07	Yes	105	90	52.3	53.6	80	81.1	STEMI	0	0	In-hospital	34.3	31.1	N.A.	manual compression	manual compression	0	1.1
**Hou et al. (18)**	2010	2005-08	No	100	100	66.2	64.9	69	72	STEMI	0*	0*	30-day	20	28	Tirofiban	manual compression	TR-Band	0	4
**Mann et al. (19)**	1998	1997	No	77	65	62	63	78	65	STEMINSTEMIUA	N.A.	N.A.	In-hospital	10	15	Abciximab	manual compression + FemoStop	radial artery compression device	0	13.8
**RADIAL-AMI (20)**	2005	N.A.	Yes	25	25	58	52	0	76	STEMI	68	64	30-day	92	95	Abciximab Tirofiban Eptifibatide	manual compression (92%). VCD (8%).	manual compression	0	4
**RADIAMI (21)**	2009	2005-06	No	50	50	59.1	59.9	48.5	51.5	STEMI	0	0	In-hospital	42	44	Abciximab	manual compression	TR-Band	2	8
**RADIAMI II (22)**	2011	2006-08	No	59	49	57.6	62.1	63	65	STEMI	0*	0*	In-hospital	54	51	Abciximab	StarClose device	TR-Band	1.7	4.1
**RIFLE-STEACS (23)**	2012	2009-11	Yes	501	500	65	65	71.9	74.8	STEMI	7	8.2	30-day	69.9	67.4	N.A.	N.A.	N.A.	2.8	9.6
**RIVAL (24)**	2011	2006-10	Yes	3,514	3,507	62	62	72.9	74.1	STEMINSTEMIUA	11.1	10.6	30-day	24	25.3	N.A.	manual compression (74.4%). VCD (25.6%)	N.A.	0.9	7
**TEMPURA (25)**	2003	1999-2001	No	72	77	67	66	81.9	80.5	STEMI	0*	0*	In-hospital	0†	0†	-	manual compression	manual compression	0	1.5
**Wang et al. (26)**	2012	2008-10	No	59	60	60.2	59.8	83.1	86.7	STEMI‡	N.A.	N.A.	In-hospital	50.8	55	Tirofiban	manual compression	manual compression	1.7	6.7

* Only patients treated with primary PCI were enrolled.

† Glycoprotein IIb/IIIa inhibitors were not given in any of the patients because not approved for clinical use in Japan.

‡ STEMI patients receiving routine early PCI within 12 hours after thrombolysis were enrolled.

PCI, Percutaneous coronary intervention; ACS, Acute coronary syndrome; STEMI, ST-segment elevation myocardial infarction; NSTEMI, Non-ST-segment elevation myocardial infarction; UA, Unstable angina; TFA, Transfemoral approach; TRA, Transradial approach; VCD, vascular closure devices; N.A., Not available data.

**Table 2:** Risk of bias assessment.

Trial Name	Random sequence generation	Allocation concealment	Blinding of participants and personnel	Blinding of outcome assessment	Incomplete outcome data	Selective reporting	Other bias	Sample size calculation
**FARMI (16)**	Unclear risk	Unclear risk	High risk	High risk	Low risk	Low risk	Low risk	No
**Gan et al. (17)**	Unclear risk	Unclear risk	High risk	High risk	Low risk	Low risk	Low risk	No
**Hou et al. (18)**	Unclear risk	Unclear risk	High risk	High risk	Low risk	Low risk	Low risk	No
**Mann et al. (19)**	Unclear risk	Unclear risk	High risk	High risk	Low risk	Low risk	High risk	No
**RADIAL-AMI (20)**	Low risk	Low risk	High risk	High risk	Low risk	Low risk	Low risk	Yes
**RADIAMI (21)**	High risk	High risk	High risk	High risk	Low risk	Low risk	Low risk	No
**RADIAMI II (22)**	High risk	High risk	High risk	High risk	Low risk	Low risk	Low risk	No
**RIFLE-STEACS (23)**	Low risk	Low risk	High risk	Low risk	Low risk	Low risk	Low risk	Yes
**RIVAL (24)**	Low risk	High risk	High risk	Low risk	Low risk	Low risk	Low risk	Yes
**TEMPURA (25)**	Unclear risk	Unclear risk	High risk	High risk	Low risk	Low risk	Low risk	Yes
**Wang et al. (26)**	Low risk	Low risk	High risk	High risk	Low risk	Low risk	Low risk	Yes

### Clinical outcomes

**Figure 2:**
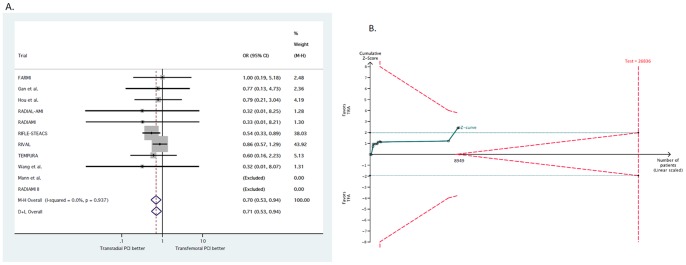
Effect of transradial vs. transfemoral approach on death. **2A.** Odds ratio of death with transradial vs. transfemoral approach. The squares and the horizontal lines indicate the OR and the 95% CIs for each trial included; the size of each square is proportional to the statistical weight of a trial in the meta-analysis; diamond indicates the effect estimate derived from meta-analysis, with the centre indicating the point estimate and the left and the right ends the 95% CI. M-H, Mantel-Hænzel model; D+L, DerSimonian and Laird model. **2B.** Trial sequential analysis for death. Heterogeneity adjusted information size of 26,836 participants calculated on basis of death of 2.53% in the transfemoral group, relative risk reduction 20%, α = 5%, β = 20%, I^2^ = 0%. Solid green cumulative Z-curve did not cross red dashed trial sequential monitoring boundaries for benefit or harm. Horizontal dotted green lines illustrate traditional level of statistical significance (p = 0.05).

**Figure 3:**
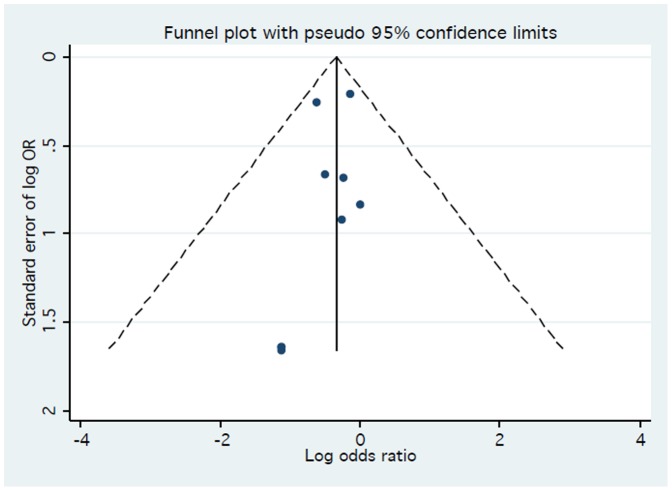
Funnel plot for the primary endpoint.

A total of 200 patients died (2.15%). As reported in Figure 2A, TRA was associated with a significant reduction in death as compared to TFA (1.81% vs. 2.53%, respectively, OR 0.70; 95% CI, 0.53–0.94; p = 0.016). No heterogeneity was found among trials (I^2^ = 0%; 95% CI, 0–65%; p_het_ = 0.94). Visual inspection of funnel plot did not reveal a skewed distribution for death, suggesting the absence of small-study effects (Figure 3). Moreover, both Harbord (p = 0.93) and Peters tests (p = 0.18) were not significant. The NNT to prevent one death with TRA was 136.3 and 7.3 (95% CI, 1.6–11.7) deaths were prevented in each 1,000 patients treated; these data were based on an OR = 0.70 applied to the control group event rate.

Trial sequential analysis showed a lack of sufficient evidence of a benefit of TRA for the reduction of death. Only the 33% (8,949 out of 26,836) of the required sample size was accrued to detect a 20% relative risk reduction for death (Figure 2B).

**Figure 4:**
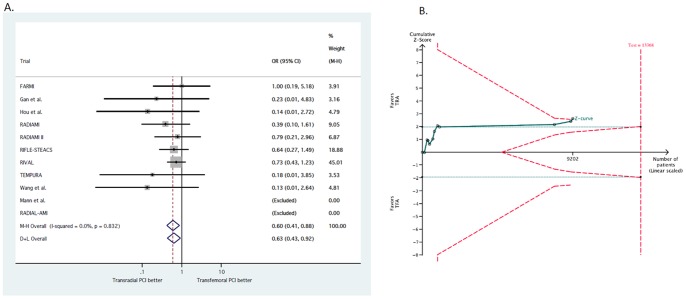
Effect of transradial vs. transfemoral approach on major bleeding. **4A**. Odds ratio of major bleeding with transradial vs. transfemoral approach. **4B**. Trial sequential analysis for major bleeding. Heterogeneity adjusted information size of 13,368 participants calculated on basis of major bleeding of 1.58% in the transfemoral group, relative risk reduction 35%, α = 5%, β = 20%, I^2^ = 0%. Solid green cumulative Z-curve crossed both red dashed trial sequential monitoring and information size boundaries, thereby confirming that transradial approach is superior to transfemoral approach in reducing vascular complications. Horizontal dotted green lines illustrate traditional level of statistical significance (p = 0.05).

Major bleeding was reported in a total of 116 patients (1.25%). As shown in Figure 4A, TRA significantly reduced major bleeding complications as compared with TFA (0.94% vs. 1.58%, respectively, OR 0.60; 95% CI, 0.41–0.88; p = 0.008). No heterogeneity was found across trials (I^2^ = 0%; 95% CI, 0–65%; p_het_ = 0.83). The NNT to prevent one major bleeding with TRA was 160.8 and 6.2 (95% CI, 1.9–9.2) major bleedings were prevented in each 1,000 patients treated; these data were based on an OR = 0.60 applied to the control group event rate.

In trial sequential analysis, despite the required information size was not met (13,368 patients), the cumulative Z-curve crossed the trial sequential monitoring boundary, indicating that sufficient evidence exists for a 35% reduction in the relative risk of major bleeding with TRA (Figure 4B).

**Figure 5:**
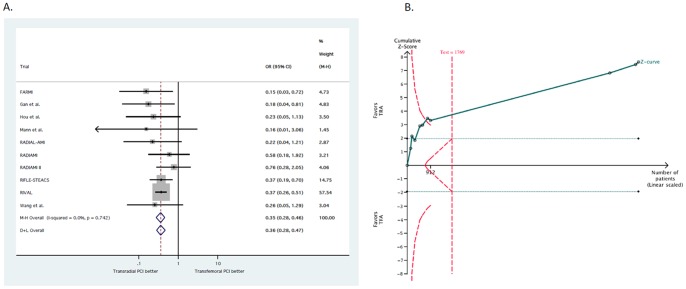
Effect of transradial vs. transfemoral approach on vascular complications. **5A**. Odds ratio of vascular complications with transradial vs. transfemoral approach. **5B**. Trial sequential analysis for vascular complications. Heterogeneity adjusted information size of 1,769 participants calculated on basis of major bleeding of 5.12% in the transfemoral group, relative risk reduction 50%, α = 5%, β = 20%, I^2^ = 0%. Solid green cumulative Z-curve crossed the red dashed monitoring boundaries, demonstrating sufficient evidence reached for 35% reduction in the risk of major bleeding with transradial approach. Horizontal dotted green lines illustrate traditional level of statistical significance (p = 0.05).

Data about vascular complications were available for 9,053 patients (98%). A total of 318 patients (3.51%) had vascular complications. As reported in Figure 5A, TRA was associated with a significant reduction in vascular complications (1.88% vs. 5.12% OR 0.35; 95% CI, 0.28–0.46; p<0.001). No heterogeneity was found across trials (I^2^ = 0%; 95% CI, 0–62%; p_het_ = 0.74). The NNT to prevent one vascular complication with TRA was 30.8 and 32.4 (95% CI, 27.1–36.6) vascular complications were prevented in each 1,000 patients treated; these data were based on an OR = 0.35 applied to the control group event rate.

Trial sequential analysis confirmed that TRA is superior to TFA in reducing vascular complications (Figure 5B).

### Influence analysis and meta-regression

Influence analysis demonstrated that no single study significantly altered the summary ORs for the endpoints, because one at a time study omission did not result in a movement of the point estimate outside the 95% CI (Figures S1–S3 in File S1).

**Figure 6:**
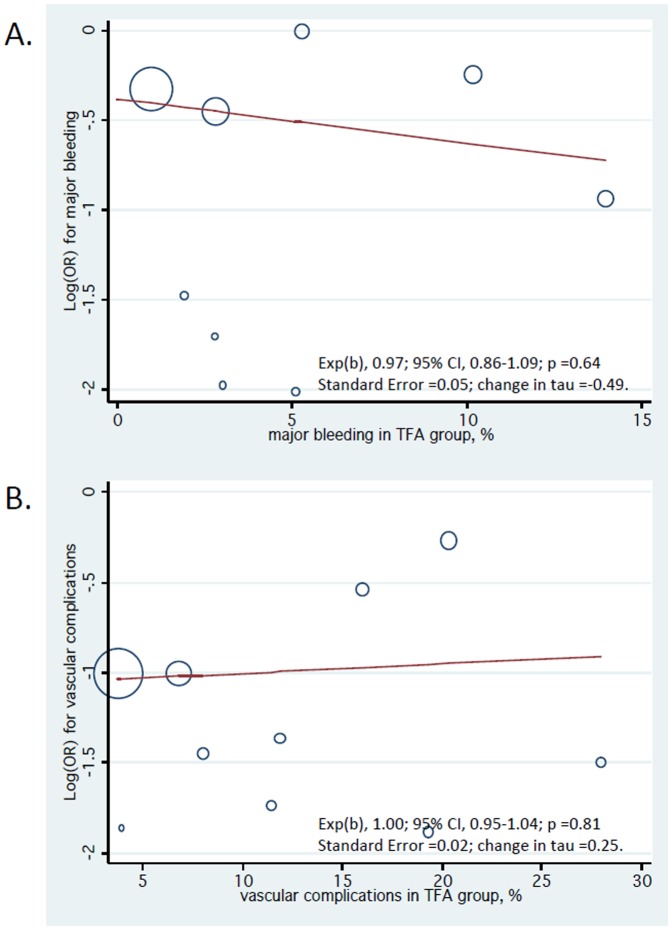
Meta-regression analysis for major bleeding (A) and vascular complications (B). The size of circles is proportional to the weight of each study in the fitted random-effects meta-regression. TFA, transfemoral.

None of the study-level covariates significantly influenced the risk of study endpoints at meta-regression analysis (Table S2 in File S1). Furthermore, we did not find a significant relationship between TRA-related risk reduction in major bleeding and major bleeding events in the TFA population (p = 0.64), as well as TRA-related risk reduction in vascular complications and the vascular complication events in the TFA population (p = 0.81) (Figure 6).

## Discussion

The main findings of this study are that: TRA compared with TFA reduced the risk of major bleeding and vascular complications in patients with ACS; despite the meta-analysis showed a reduced risk of death with TRA, there is no definite evidence supporting this association as demonstrated by trial sequential analysis; the meta-regression analysis suggested that the benefit with TRA, in terms of bleeding and vascular complications, was irrespective of the patient risk profile.

The incidence of recurrent ischemic events in patients with ACS has been drastically reduced by the combination of multiple antithrombotic agents, along with the early use of coronary angiography with a view to revascularization [27]. Nevertheless, the benefit derived from these therapies led to an increase in the rates of bleeding complications. In the recent years, several studies demonstrated that bleeding occurrence in patients undergoing PCI is associated with a worse prognosis in terms of death, myocardial infarction, and stroke [28]. At this regard, TRA has the potential to decrease bleeding events, primarily by reducing vascular complications [29]. The main results of this study including 11 randomized trials with a total of 9,202 patients were in accordance with previous meta-analyses that dealt exclusively with patients with ST-elevation myocardial infarction [5], [6], [30]. However, these meta-analyses were limited by the inclusion of the ST-elevation myocardial infarction subgroup of the RIVAL trial [24], in which randomization was not stratified by clinical presentation.

Despite we found a 30% reduction in the odds of death in favour of TRA, with about 7 deaths prevented per 1,000 patients treated, this finding was not confirmed in trial sequential analysis, which disclosed an insufficient evidence for this association. Indeed, current available data do not conclusively support a 20% relative risk reduction in mortality with TRA, since only one third of the required study population was accrued in this study. Thus, trial sequential analysis should be implemented when a meta-analysis is performed, since many apparently conclusive meta-analyses may become inconclusive when the statistical analyses take into account the risk of random error due to repetitive testing.

In contrast with previous meta-analyses [5], [6], we reported for the first time a significant reduction in the risk of major bleeding in TRA group, attributable to the higher statistical power of this study. Trial sequential analysis showed a sufficient evidence for this association, despite the required information size was not reached (9,202 out of 13,368 patients). At this regard, a possible advantage of this methodological tool is that it may prevent the initiation of unnecessary trials when firm evidence has been gained [13].

The decreased risk of major bleeding, along with the dramatic reduction in vascular complications, might provide an explanation to the possible risk reduction in death. In this respect, a large pooled analysis of three randomized trials including ACS patients found that approximately 1 in 10 patients who developed major bleeding died during the first 30 days after hospitalization compared with 1 in 40 of those who did not develop major bleeding [31]. Consistently, an analysis of the ACUITY (Acute Catheterization and Urgent Intervention Triage strategy) trial showed that both major bleeding and myocardial infarction have a similar association with mortality, carrying a similar risk of death in the first year following presentation with an ACS [32]. In addition, Doyle et al. found that patients experiencing major femoral bleeding after PCI had a higher mortality at long-term follow-up, due to an excess of death during the first 30 days [33]. However, the relationship between major bleeding after PCI and death is likely to be multifactorial. Major bleeding could directly increase the risk of death by causing hemodynamic compromise and could lead clinicians to discontinue antithrombotic agents. Bleeding also may reduce oxygen delivery to the myocardium and anaemia-induced erythropoietin release may promote a systemic prothrombotic state. Furthermore, despite increased haemoglobin levels, transfusion does not increase tissue oxygenation and its use is associated with a poor outcome in PCI patients [34]. Several non-randomized studies supported the association between bleeding reduction with TRA and mortality. In the PRESTO ACS (Comparison of Early Invasive and Conservative Treatment in Patients with Non-ST-Elevation Acute Coronary Syndromes) vascular substudy [35], TRA compared with TFA was associated with a decrease in bleeding complications and death or reinfarction at 1-year. Accordingly, a recent analysis of the HORIZONS-AMI (Harmonizing Outcomes with RevascularIZatiON and Stents) trial demonstrated a significant reduction in major bleeding and in the composite of death or reinfarction at 30-day with TRA compared with TFA [36].

Meta-regression analysis showed consistent results in single- versus multi-centre trials. This was an important finding since single-centre trials are usually associated with a larger treatment effects than multi-centre trials [37]. In addition, we found that TRA may decrease the risk of major bleeding and vascular complications independently from the patient risk profile, in a “one-size fits-all” manner (Figure 6). Although these findings should be considered hypothesis generating, they may be due to the fact that vascular access-related complications are almost eliminated with TRA, as also supported by trial sequential analysis. Accordingly, access site bleeding is the most common source of bleeding complications in patients undergoing PCI and it is associated with a 2-fold increase in 1-year mortality [38]. However, the reduction in the risk of major bleeding did not lead to a parallel risk reduction of death, probably because access-site bleeding is associated with a lower risk of death than non-access-site bleeding. In fact, among more than 3 millions of patients included in the Cath PCI Registry [39], in-hospital mortality was 2.73% vs. 1.87% for access-site vs. no bleeding, and 8.25% vs. 1.87% for non-access-site vs. no bleeding. However, none of the included trials used contemporary transfemoral closure systems that are known to consistently reduce vascular complications by more than 50% [40]. At this regard, the ARISE (AngioSeal versus the Radial approach In acute coronary SyndromE) trial will help to define the role of a vascular closure device as a bleeding avoidance strategy in patients with ACS [41]. A final remark about vascular complications is related to sheath size used for both TRA and TFA. In fact, in the Leipzig registry [42], radial artery occlusion was documented with vascular ultrasound in 30.5% and 13.7% of patients treated with 6-F and 5-F sheaths, respectively. Similar results may be achieved through 5-F TFA [43].

### Study limitations

First, this is a meta-analysis at the study level and we could not properly assess the role of confounding factors. Second, all included trials were performed by experienced operators skilled in TRA, thus limiting the external validity of the results of this meta-analysis for centres mainly performing transfemoral procedures. This reinforces the need for dedicated training programs for TRA, especially in teaching hospitals [44]. On the other hand, patients undergoing PCI from the TFA by default radial operators may be at higher risk of vascular access-site complication [45]. Third, this meta-analysis provides clinical follow-up within 30 days and it is still underpowered to evaluate the risk of death as demonstrated by trial sequential analysis. Thus, larger trials and longer follow-up data are needed to establish whether the observed benefit in mortality persists over time. Fourth, because of the frequent use of glycoprotein IIb/IIIa inhibitors in the included studies, it remains largely unknown whether the use of bivalirudin, that has been associated with lower bleeding events [46], may offset the greater risk of bleeding and vascular complications associated with TFA. Fifth, despite crossover rates to TFA did not influence study endpoints, the effect of crossover to TFA in patients undergoing primary PCI remains uncertain. At this regard, despite the risk of mortality with TRA is reduced even with longer door-to-balloon times [47], caution should be exercised in centres transitioning from a routine TFA to TRA in primary PCI setting. Six, patients with cardiogenic shock or hemodynamic instability were excluded from the most of trials. Therefore, the potential benefits of TRA in this setting are not well established. Finally, clinical data in patients with non-ST-elevation ACS derive from the RIVAL trial [24], in which the benefit of TRA was not manifest in this subgroup. Thus, further research with dedicated, adequately powered trials is needed to establish whether the benefit of TRA over TFA can be extended to patients with non-ST-elevation ACS.

### Conclusions

This study demonstrated that the use of TRA in patients with ACS undergoing invasive management was associated with a significant reduction in the risk of major bleeding and vascular complications, as compared with TFA. The robustness of these findings was confirmed by trial sequential analysis. The clinical benefit, in terms of major bleeding or vascular complications, might translate in a lower risk of death with TRA. Nevertheless, the association between mortality and TRA in ACS setting should be interpreted cautiously because it is based on insufficient evidence, as demonstrated by trial sequential analysis.

However, because of the clinical relevance associated with major bleeding and vascular complications reduction, TRA should be recommended as first-choice vascular access in patients with ACS undergoing cardiac catheterization.

## Supporting Information

File S1 (DOC)

Checklist S1 (DOC)
