# Supplementary material for: Transradial versus Transfemoral Approach in Patients Undergoing Percutaneous Coronary Intervention for Acute Coronary Syndrome. A Meta-Analysis and Trial Sequential Analysis of Randomized Controlled Trials
Source: PLoS One. 2014 May 12;9(5):e96127. doi: 10.1371/journal.pone.0096127 (PMC4018335; doi:10.1371/journal.pone.0096127)
Supplement: File S1 — (DOC) [file pone.0096127.s001.doc]

**Supporting Information**

**Transradial versus transfemoral approach in patients undergoing percutaneous coronary intervention for acute coronary syndrome.**

**A meta-analysis and trial sequential analysis of randomized controlled trials.**

**Search strategy.**

**Medline (PubMed).**

((radial [all fields] OR transradial [all fields]) AND ((femur [mh] OR femur [all fields] OR femoral [all fields]) OR transfemoral [all fields]) AND (heart [mh] OR heart [all fields] OR coronary [all fields])) AND (randomized controlled trial [pt] OR randomized controlled trial [mh] OR random allocation [mh] OR double-blind method [mh] OR single-blind method [mh] OR clinical trial [pt] OR clinical trials [mh] OR (“clinical trial” [tw]) OR ((singl* [tw] OR doubl* [tw] OR tripl* [tw]) AND (mask* [tw] OR blind* [tw])) OR random* [tw] OR comparative study [pt] OR follow-up studies [mh] OR (clinical [tw] AND (outcome* [tw] OR follow* [tw] OR result* [tw]))) AND (Acute Coronary Syndrome [mh] OR (“acute coronary syndrome*” [all fields]) OR Unstable Angina [mh] OR (“unstable angina” [all fields]) OR “ST-elevation” [tw] OR “ST-segment” [tw] OR STEMI [all fields] OR NSTEMI [all fields] OR “myocardial infarction” [all fields])

**The Cochrane Library.**

1. (transradial in All Text or trans-radial in All Text)
2. (radial in All Text)
3. (transfemoral in All Text or trans-femoral in All text)
4. (femoral in All Text)
5. MeSH descriptor Angioplasty explode all trees
6. MeSH descriptor Coronary Artery Disease explode all trees
7. (coronary in All Text)
8. (PCI in All Text or “percutaneous coronary intervention” in All Text)
9. (PTCA in All Text or “percutaneous transluminal coronary angioplasty” in All Text)
10. MeSH descriptor Randomized Controlled Trial explode all trees
11. MeSH descriptor Random Allocation explode all trees
12. (Random* in All Text)
13. (clinical in All Text near/3 outcome* in All Text)
14. (clinical in All Text near/3 follow* in All Text)
15. (clinical in All Text near/3 result* in All Text)
16. ((#1 or #2) and (#3 or #4))
17. (#5 or #6 or #7 or #8 or #9)
18. (#16 and #17)
19. (#10 or #11 or #12)
20. (#13 or #14 or #15)
21. (#16 and #19)
22. (#16 and #20)
23. (#18 and #19 and #20)
24. MeSH descriptor Acute coronary Syndrome explode all trees
25. Mesh descriptor Unstable Angina explode all trees
26. Unstable Angina in All Text
27. (“ST-elevation” in All Text or “ST-segment” in All Text or STEMI in All Text)
28. (NSTEMI in All Text)
29. (“myocardial infarction” in All Text)
30. (#24 or #25 or #26 or #27 or #28 or #29)
31. (#16 and #30)
32. (#18 and #30)

**Tables.**

**Table S1.** Endpoints definitions.

| **Trial Name** | **Major bleeding complications** | **Vascular complications** |
| --- | --- | --- |
| FARMI | TIMI major bleeding: a hemoglobin drop of >5 g/dl or intracranial hemorrhage or cardiac tamponade. | Groin haematoma: a local induration of >4 cm diameter. Ecchymosis: cutaneous bruise or induration of <4 cm diameter, or both. |
| Gan et al. | Not defined in the methods. | Not defined in the methods. |
| Hou et al. | Not defined in the methods. | Major access site bleeding: hemoglobin loss of at least 2 mmol/l, administration of blood transfusions, and needing vascular repair. Minor access site bleeding: hematoma formation not requiring specific therapy. |
| Mann et al. | Not defined in the methods. | Access site complications: a bleeding vascular complication that prolonged hospitalization. |
| RADIAL-AMI | Major bleeding: intracranial or retroperitoneal bleeding, a drop in hemoglobin level >5 g/dL or hematocrit ≥15%, or whole blood or packed red cell transfusions. | Access site complications: hematoma >5 cm, pseudoaneurysm, arteriovenous fistula, access site bleeding after initial hemostasis. |
| RADIAMI | Fatal bleeding, bleeding requiring blood transfusion, operation or resulting in a drop of hemoglobin count of more than 3 g/dL as well as any intracranial hemorrhage. | Not defined in the methods. |
| RADIAMI II | Bleeding that resulted in death or a need for blood transfusion or surgical intervention, caused hemoglobin level decrease by >3 g/dL, and central nervous system bleedings. | Not defined in the methods. |
| RIFLE-STEACS | Any overt and actionable hemorrhage not related to coronary artery bypass graft with ≥3 g/dl decrease in hemoglobin, requiring prompt evaluation by a health care professional and leading to an increased level of care. | Bleeding was categorized as access site and non–access site related according to its relationship to the arterial vascular access. |
| RIVAL | Bleeding that was fatal, resulted in transfusion of two or more units of red blood cells or equivalent whole blood, caused substantial hypotension with the need for inotropes, needed surgical intervention (a requirement for surgical access site repair constitutes major bleeding only if there has been substantial hypotension or transfusion of at least two units of blood), caused severely disabling sequelae, was intracranial and symptomatic or intraocular and led to significant visual loss, or led to a drop in hemoglobin of at least 50 g/L. | Pseudoaneurysm requiring closure, arterio-venous fistula, large hematoma (as judged by investigator), ischemic limb requiring surgery. |
| TEMPURA | Bleeding requiring blood transfusion and/or surgical repair or cerebral bleeding. | Vascular complications were not evaluated in this study. |
| Wang et al. | Hemoglobin loss of at least 2 g/L, the administration of a blood transfusion, vascular repair, or prolonged hospitalization, but minor vascular access-site bleeding was defined as hematoma formation (hematoma <5 cm in diameter) not requiring specific therapy. | Not defined in the methods. |

**Table S2. Meta-regression analysis.**

| **Variable** | **Exp(b)** | **95%CI** | **SE** | **Change**  **in tau** | **p-value** |
| --- | --- | --- | --- | --- | --- |
| **Mean age** (years) |  | | | | |
| Death | 0.95 | 0.84-1.10 | 0.05 | -0.71 | 0.50 |
| Major Bleeding | 1.00 | 0.81-1.22 | 0.08 | 0.00 | 0.99 |
| Vascular complications | 1.02 | 0.90-1.15 | 0.05 | 0.46 | 0.65 |
| **Sex** (percentage of males) |  | | | | |
| Death | 1.01 | 0.94-1.09 | 0.03 | 0.51 | 0.62 |
| Major Bleeding | 1.00 | 0.95-1.07 | 0.03 | 0.30 | 0.77 |
| Vascular complications | 0.98 | 0.94-1.02 | 0.01 | -1.00 | 0.35 |
| **Year of publication** |  | | | | |
| Death | 0.99 | 0.83-1.18 | 0.07 | -0.07 | 0.95 |
| Major Bleeding | 1.07 | 0.79-1.44 | 0.13 | 0.53 | 0.61 |
| Vascular complications | 1.08 | 0.91-1.29 | 0.08 | 1.09 | 0.31 |
| **Enrolling centres** (single- vs. multi-centre) |  | | | | |
| Death | 1.02 | 0.36-2.90 | 0.45 | 0.06 | 0.95 |
| Major Bleeding | 1.37 | 0.48-3.94 | 0.61 | 0.72 | 0.49 |
| Vascular complications | 0.87 | 0.40-1.85 | 0.28 | -0.43 | 0.68 |
| **Sample size** (<150 patients vs. ≥150 patients) |  | | | | |
| Death | 1.18 | 0.36-3.83 | 0.58 | 0.34 | 0.74 |
| Major Bleeding | 1.21 | 0.41-3.57 | 0.55 | 0.43 | 0.68 |
| Vascular complications | 0.86 | 0.40-1.85 | 0.28 | -0.44 | 0.67 |
| **Use of glycoprotein IIb/IIIa inhibitors** (%) |  | | | | |
| Death | 0.99 | 0.98-1.01 | 0.00 | -1.03 | 0.34 |
| Major Bleeding | 1.00 | 0.98-1.02 | 0.00 | 0.30 | 0.77 |
| Vascular complications | 0.99 | 0.98-1.01 | 0.00 | -0.35 | 0.73 |
| **Primary percutaneous coronary intervention** (%) |  | | | | |
| Death | 0.99 | 0.98-1.00 | 0.00 | -1.23 | 0.26 |
| Major Bleeding | 0.99 | 0.98-1.00 | 0.00 | -0.64 | 0.54 |
| Vascular complications | 1.00 | 0.99-1.01 | 0.00 | 0.59 | 0.57 |
| **Crossover rates to TFA** (%) |  |  |  |  |  |
| Death | 0.97 | 0.84-1.14 | 0.06 | -0.33 | 0.75 |
| Major Bleeding | 1.07 | 0.86-1.33 | 0.09 | 0.75 | 0.48 |
| Vascular complications | 0.97 | 0.84-1.13 | 0.06 | -0.37 | 0.72 |

CI, confidence intervals, SE, standard error; TFA, transfemoral approach.

**Figures.**

**Figure S1.** Influence analysis for death.

**Figure S2.** Influence analysis for bleeding events.

**Figure S3.** Influence analysis for vascular complications.
